# Effect of inspiratory lung volume on bronchial and arterial dimensions and ratios on chest computed tomography in patients with chronic obstructive pulmonary disease

**DOI:** 10.1007/s00330-024-11126-3

**Published:** 2024-11-29

**Authors:** Yuxin Chen, Rudolfs Latisenko, David A. Lynch, Pierluigi Ciet, Jean-Paul Charbonnier, Harm A. W. M. Tiddens

**Affiliations:** 1 Division of Respiratory Medicine and Allergology, Department of Paediatrics, Sophia Children’s Hospital, Erasmus MC, Rotterdam, The Netherlands; 2 Department of Radiology and Nuclear Medicine, Erasmus MC, Rotterdam, The Netherlands; 3 Thirona, Nijmegen, The Netherlands; 4 Department of Radiology, National Jewish Health, Denver, CO USA; 5 Department of Radiology and Medical Science, University of Cagliari, Cagliari, Italy

**Keywords:** Lung volume, Bronchus and artery, Airway disease, Inspiratory, Tomography (X-ray computed)

## Abstract

**Background:**

The assessment of bronchus–artery (BA) metrics on chest CT is important for detecting airway abnormalities. It is less clear how BA metrics are dependent on lung volume.

**Methods:**

CTs were obtained from a COPDGene substudy investigating the impact of radiation dose on lung density. Patients with chronic obstructive pulmonary disease underwent a full-dose and a reduced-dose CT in the same imaging session. CTs were automatically analyzed by measuring diameters of the bronchial outer edge (*B*_out_), bronchial inner wall (*B*_in_), artery (*A*), and bronchial wall thickness (*B*_wt_) from segmental (*G*_0_) and distal generations. BA ratios were computed: *B*_out_/*A*, *B*_in_/*A*, *B*_wt_/*A*, and bronchial wall area/bronchial outer area (*B*_wa_/*B*_oa_). The total lung volume of the CT (TLC-CT) was computed. Differences between the volumes between the two CTs were expressed as % of the highest TLC-CT (ΔTLC-CT%). For the BA metrics of each CT, we computed the median of measurements in G_1–6._ Mixed-effect models were used to investigate the influence of TLC-CT on BA metrics adjusted for dose protocol.

**Results:**

One thousand three hundred nineteen patients with a mean (SD) age of 64.4 (8.7) years were included. Three hundred twenty-nine (124) BA pairs were analyzed per CT. No significant difference was found for TLC-CT in relation to dose (*p* = 0.17). A ΔTLC-CT% of >10% (found in 121, 9%) led to 0.03 and 0.05 decreases in *B*_out_/*A* and *B*_in_/*A* and 0.008 and 0.11 decrease in log (*B*_wt_/*A*) and log (*B*_wa_/*B*_oa_), and a 0.03 increase in *B*_in_ and 0.06, 0.12, and 0.04 decrease in *B*_out_, log (*B*_wt_), and log (*A*) (all *p* < 0.001).

**Conclusions:**

Variations in TLC over 10% between time points significantly influence bronchial dimensions, affecting BA metrics. Standardizing volumes is recommended for sensitive tracking of airway disease changes over time.

**Key Points:**

***Question*** *Are BA metrics dependent on total lung capacity (TLC), and if so, how*?

***Findings*** *TLC variations over 10% between time points significantly influence bronchial dimensions, affecting BA metrics. Variations below 10% between CT scans have little effect on BA metrics*.

***Clinical relevance*** *Small lung volume differences between chest CTs have little impact on bronchus and artery metrics; it is imperative to standardize chest CT lung volumes to ensure precise diagnosis and monitoring of airway disease*.

## Introduction

Chronic obstructive pulmonary disease (COPD) is characterized by progressive and irreversible airflow obstruction due to airway remodeling and emphysema. Lung pathology studies in COPD have observed a reduced number of small airways, narrowed terminal bronchioles, and thickened bronchial walls [1, 2]. Furthermore, it has been shown that geometrical changes in more central airways are correlated with changes in the small airways [3]. The manual measuring of airway dimensions on chest CTs is extremely time-consuming and therefore not feasible in clinical practice [4, 5]. Quantitative artificial intelligence (AI) based CT analysis in COPD now allows for the sensitive and accurate measurements of central bronchial dimensions, which are relevant for the detection and monitoring of structural airway changes. The consequences of small airway disease can be detected indirectly as low attenuation regions on expiratory scans. In COPD, these regions reflect the combination of trapped air, emphysema, and hypoperfusion [6]. Furthermore, emphysema is an important structural change of the lung parenchyma in COPD. Emphysema is likely to influence the bronchial dimensions of especially the smaller airways in relation to lung volume [7]. In clinical practice, chest CT scanning is a sensitive imaging modality to assess bronchial remodeling. Radiologists assess bronchial remodeling by visually comparing the dimensions of the bronchus to those of the adjacent pulmonary artery. In general, the clinical definition for bronchiectasis is when the ratio of the inner diameter of the bronchus and artery (BA) exceeds 1.0 [8, 9]. However, for the visual detection of bronchiectasis, a more conservative ratio between the inner or outer diameter of the BA of 1.5 has been suggested [10]. Though objective measurements of BA dimensions and ratios of all visible BA pairs would be preferred this is currently not clinical practice as manual measurements are extremely time-consuming. Recently, an artificial intelligence-based algorithm has been developed that is capable of precisely and automatically measuring the BA dimensions of a large number of BA pairs on chest CT scans. This BA method has been validated in various cohorts, including subjects with normal chest CTs, severe asthma, bronchiectasis, and cystic fibrosis [4, 5, 11, 12].

It is well known that the bronchial diameters are dependent on the inspiratory lung volume at which the chest CT scan is conducted [4, 7, 11, 13–17]. BA ratios were found to be dependent on lung volume as has been shown in various populations, including children with cystic fibrosis [4, 15], adults with COPD [13, 18], and subjects with normal chest CTs [14]. The pulmonary artery diameter, on the other hand, is generally considered to remain relatively constant in diameter throughout the breathing cycle. However, to the best of our knowledge, there are no studies to support this. For this reason, it has been advocated that the lung volume for inspiratory chest CT scans used for the assessment of BA metrics should be acquired near total lung capacity (TLC) and that this procedure should be standardized [19–21]. However, the specific inspiratory lung volumes that remain acceptable for accurate assessment of BA metrics have yet to be firmly established [15]. The aim of this study was to investigate the effect of lung volume on BA dimensions and ratios in patients with COPD participating in the Genetic Epidemiology of COPD (COPDGene) cohort. We hypothesized that inspiratory lung volume influences both BA dimensions and ratios.

## Methods

### Study population

The COPDGene study (ClinicalTrials.gov: NCT00608764) [22] is a prospective multicenter observational cohort study of more than 10,000 individuals of current and former smokers at the time of inclusion. The COPDGene study was approved by the institutional review boards at each of the 21 participating clinical sites. Written informed consent was obtained from all participants [23].

### CT imaging

For our study, we obtained Chest CTs from patients who had previously participated in a COPDGene substudy. One thousand two hundred-five of the 1319 patients have been previously reported [23]. This prior article was aimed to investigate the influence of radiation dose on lung density whereas in this manuscript we report on the effect of inspiratory lung volume on BA metrics. In this substudy, all patients underwent two CT scans in the same imaging session: a standard full-dose (FD; 120 kVp, 200 mAs) and a reduced-dose (RD; 120 kVp, 35 mAs) scan. Detailed CT protocols have been reported in the previous publication [23]. The FD and RD scans were performed during consecutive voluntary inspiratory breath-holds without the patient leaving the CT gantry. To ensure consistent and optimal breath-hold volume, site CT technicians followed a standardized set of breathing instructions, with the goal of achieving a breath-hold volume exceeding 90% of predicted TLC. The specific breathing instructions can be found in the Appendix. The acquisition of these two CTs, performed within a short time interval, allowed us to investigate the relationship between lung volume on BA dimensions and ratios.

### Imaging analysis

Total lung capacity measured on chest CT (TLC-CT) and BA-metrics were analyzed on chest CTs using LungQ (v2.1.0.1, Thirona). LungQ automatically segments the bronchial tree, identifies the segmental (*G*_0_) and distal bronchial generations, and measures the following BA dimensions: bronchial outer wall diameter (*B*_out_), inner wall diameter (*B*_in_), bronchial wall thickness (*B*_wt_), and artery diameter (*A*) adjacent to the bronchus. The bronchi quantification utilizes a proprietary intensity profile quantification algorithm that allows for sub-resolution quantification of *B*_wt_. For each bronchial generation *G*_0_ and higher, the BA dimensions of each individual bronchial branch are computed as the average of all measurements within that branch. From these dimensions, the following BA ratios are computed: *B*_out_/*A*, *B*_in_/*A*, *B*_wt_/*A*, and *B*_wa_/*B*_oa_ (= bronchial wall area/bronchial outer area). TLC-CT measurements are expressed in milliliters and as a percentage of the predicted value for TLC for each individual using the global lung function initiative (GLI) based on age, sex, and height of the study subject [24].

### Statistical analysis

For each BA metric, we computed the median of all BA dimensions and ratios in G_1–6_ for analysis [14]. Bland–Altman plots were used to evaluate the bias (mean of differences) and limits of agreement (1.96 times the standard deviation of differences) for TLC-CT and BA metrics between FD and RD scans. A paired *t*-test was performed to test the difference in TLC-CT between FD and RD scans.

To analyze the variability in TLC-CT between the highest and lowest lung volumes for FD and RD of each patient, we computed the difference (ΔTLC-CT) as a percentage of the highest lung volume CT (ΔTLC-CT%). Similarly, the differences in BA metrics were calculated by subtracting the BA metrics for the CT with the lowest TLC-CT from that of the highest TLC-CT. Boxplots were used to plot the difference in BA metrics against ΔTLC-CT%. Linear mixed-effect models were used to investigate the influence of TLC-CT on BA metrics. The model assumed TLC-CT%, dose protocol, and their interaction as fixed effects and accounted for measurements that were taken from the same patient as random effects. The interaction between TLC-CT% and dose protocol allows the effect of TLC-CT% on the outcomes to be per dose protocol. We did not include sex, age, and height as potential confounders, as they were already taken into account in the calculation of predicted TLC values for each patient. Log transformation was used for *B*_wt_/*A*, *B*_wa_/*B*_oa_, *B*_wt_, and *A*. Statistical significance was accepted for *p*-values less than 0.05. Statistical analyses were done with R (version 4.2.3).

## Results

### Study population

Two thousand six hundred thirty-eight CTs of 1319 patients with a mean (SD) age of 64.4 (8.7) years were included. Patient characteristics are summarized in Table 1. In total, 772,770 BA pairs were detected and measured. On average 329 BA pairs per CT were analyzed. 93% of BA pairs were detected in segmental generation G_1–6_. The mean (SD) lung volume of the TLC-CTs relative to predicted TLCs calculated from GLI was 86% (18%).

**Table 1:** Baseline characteristics

	*N* = 1319
Age, (years)	64.4 ± 8.7
Gender	
Male	665 (50.4%)
Female	654 (49.6%)
Height, (cm)*	169.6 ± 9.7
Weight, (Kg)*	83.61 ± 19.9
GOLD stage	
0	542 (41.1%)
1	116 (8.8%)
2	204 (15.5%)
3	80 (6.1%)
4	34 (2.6%)
PRISm	191 (14.5%)
Never smokers	128 (9.7%)
Unknown**	24 (1.8%)
Difference in TLC-CT%	
< 1%	393 (30%)
1–2.5%	342 (26%)
2.5–5%	269 (20%)
5–10%	194 (15%)
> 10%	121 (9%)

Data are mean ± standard deviation or *n* (%)

*GOLD* global initiative for chronic obstructive lung disease, *PRISm* preserved ratio impaired spirometry, *TLC-CT%* *TLC-CT* total lung capacity measured from chest CT

* Height and weight were summarized from 1318 patients

** The category GOLD stage unknown represents those COPD patients for whom no spirometry data were available

### The difference in TLC-CT between FD and RD scans

**Fig. 1:**
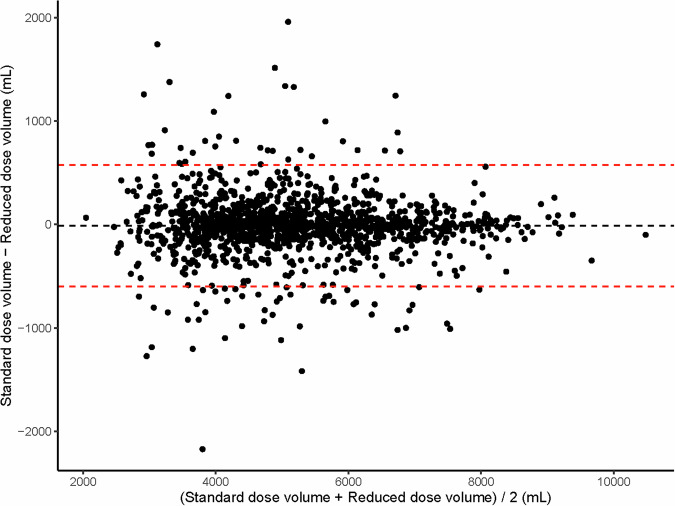
Bland–Altman plot for the difference in TLC between FD and RD CT scans. The systematic bias between the volume of the standard dose CT and the reduced dose is −11.3 mL (dotted black line). The limits of agreement (1.96*SD) are between −575.5 mL and 598.1 mL (dotted red line)

There was no significant difference in TLC-CT between FD and RD scans (*p* = 0.17). The difference in TLC-CT between FD and RD scans using a Bland–Altman plot is shown in Fig. 1. The bias (limits of agreements) was −11.3 (−575.5, 598.1) mL between FD and RD scans. The mean ΔTLC-CT% between FD and RD scans was 3.84 (4.98)%. 393 (30%) patients had a ΔTLC-CT% < 1% and 121 (9%) ΔTLC-CT% > 10% (Table 1). Seven patients had the same TLC-CT as measured on FD and RD scans. For 701/1319 (53%) patients TLC-CT_FD was less than TLC-CT_RD and for 611/1319 (46%) patients TLC-CT_FD was greater than TLC-CT_RD (Fig. S2). In patients with a ΔTLC-CT% > 10%, 57/121 (47%) patients had TLC-CT_FD less than TLC-CT_RD, and 64/121 (53%) patients had TLC-CT_FD greater than TLC-CT_RD.

### Effect of TLC-CT on BA-metrics between FD and RD scans

**Fig. 2:**
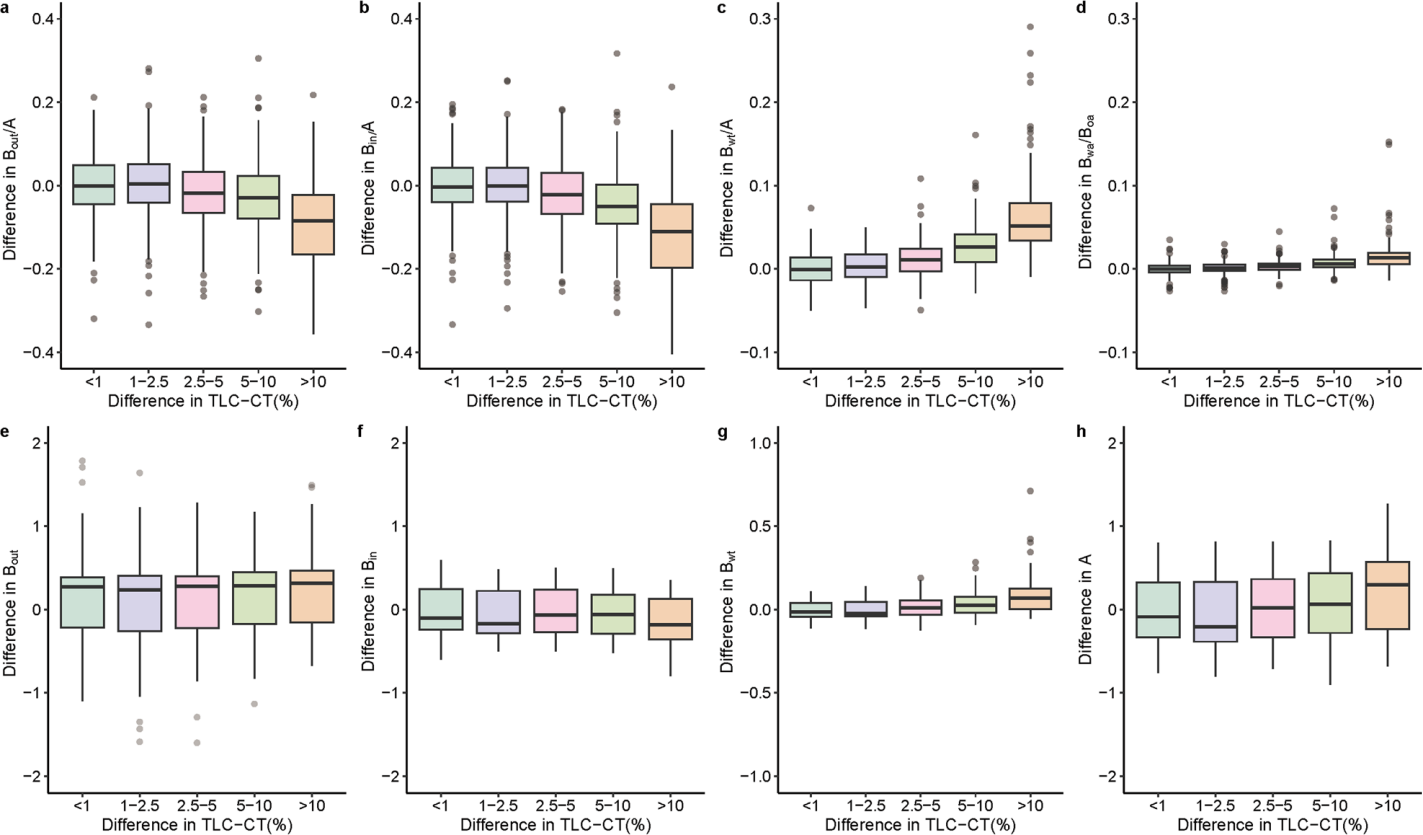
Difference in BA-metrics against difference in TLC as determined between FD and RD CT scans in all patients. The difference in BA ratios (**a**–**d**) and BA dimensions (**e**–**h**) against the difference in TLC was determined between FD and RD CT scans. The difference in TLC-CT was computed between the CT with the highest TLC-CT and lowest TLC-CT (ΔTLC-CT). This difference was expressed as a percentage of the highest lung volume CT (ΔTLC-CT%). Differences in BA metrics were calculated by subtracting the BA metrics for the CT with the lowest TLC-CT from that of the highest TLC-CT. BA, bronchus and artery; TLC-CT, total lung capacity measured from chest CT; *B*_out_, bronchial outer diameter; *B*_in_, bronchial inner diameter; *B*_wt_, bronchial wall thickness; A, artery diameter; *B*_wa_/*B*_oa_, bronchial wall area divided by bronchial outer area. Note that overall ΔTLC-CT% of < 10% had little to no effect on BA-metrics

**Fig. 3:**
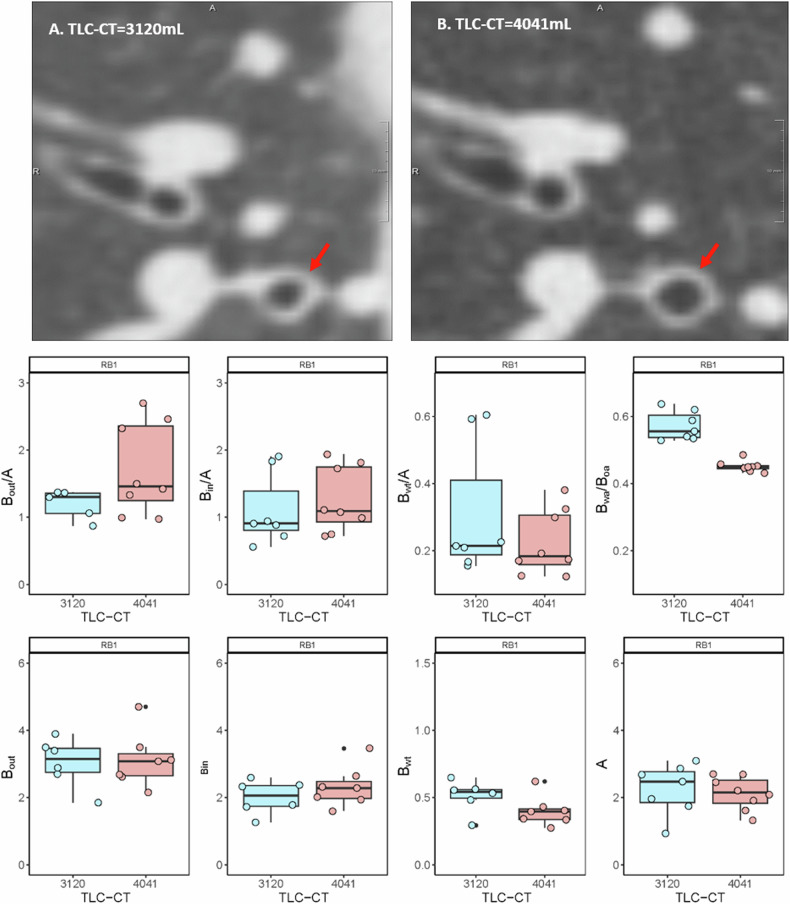
Comparison of inspiratory volume variation in RB1 through two CT slices from a single patient. The patient underwent two CT scans, one with RD imaging at an inspiratory volume of 3120 mL (**A**), and another with full-dose imaging at an inspiratory volume of 4041 mL (**B**). Both scans reveal slices in the RB1 area, highlighting significant variations within the same bronchus (red arrow). The boxplots showed the BA ratios and BA diameters for all detectable bronchi within the RB1 generation across the two CT scans. Note that as TLC-CT increased, there was an observed increase in *B*_out_/*A* and *B*_in_/*A*, while there was a decrease in *B*_wt_/*A* and *B*_wa_/*B*_oa_. BA, bronchus and artery; TLC-CT, total lung capacity measured from chest CT; *B*_out_, bronchial outer diameter; *B*_in_, bronchial inner diameter; *B*_wt_, bronchial wall thickness; A, adjacent artery diameter; *B*_wa_/*B*_oa_, bronchial wall area divided by bronchial outer area

CTs of all patients were included to assess the effect of TLC-CT on BA metrics. The difference in BA ratios against the difference in ΔTLC-CT% is plotted in Fig. 2, indicating that ΔTLC-CT% of < 10% had little to no effect on BA metrics. For the statistical model, we adjusted for the dosing protocol and found a 10% increase in lung volume significantly led to 0.03 and 0.05 increase in *B*_out_/*A* and *B*_in_/*A* and 0.008 and 0.11 decrease in log (*B*_wt_/*A*) and log (*B*_wa_/*B*_oa_) (all *p* < 0.001, Table S1)_._ For BA dimensions, a 10% increase in lung volume led to a 0.03 increase in *B*_in_ and 0.06, 0.12, and 0.04 decrease in *B*_out_, log (*B*_wt_), and log (*A*) (all *p* < 0.001, Table S1). Figure 3 shows a comparison of the impact of TLC-CT variation in the apical segment (RB1) across two CT slices from a single patient.

### Effect of dose on BA-metrics between FD and RD scans

**Fig. 4:**
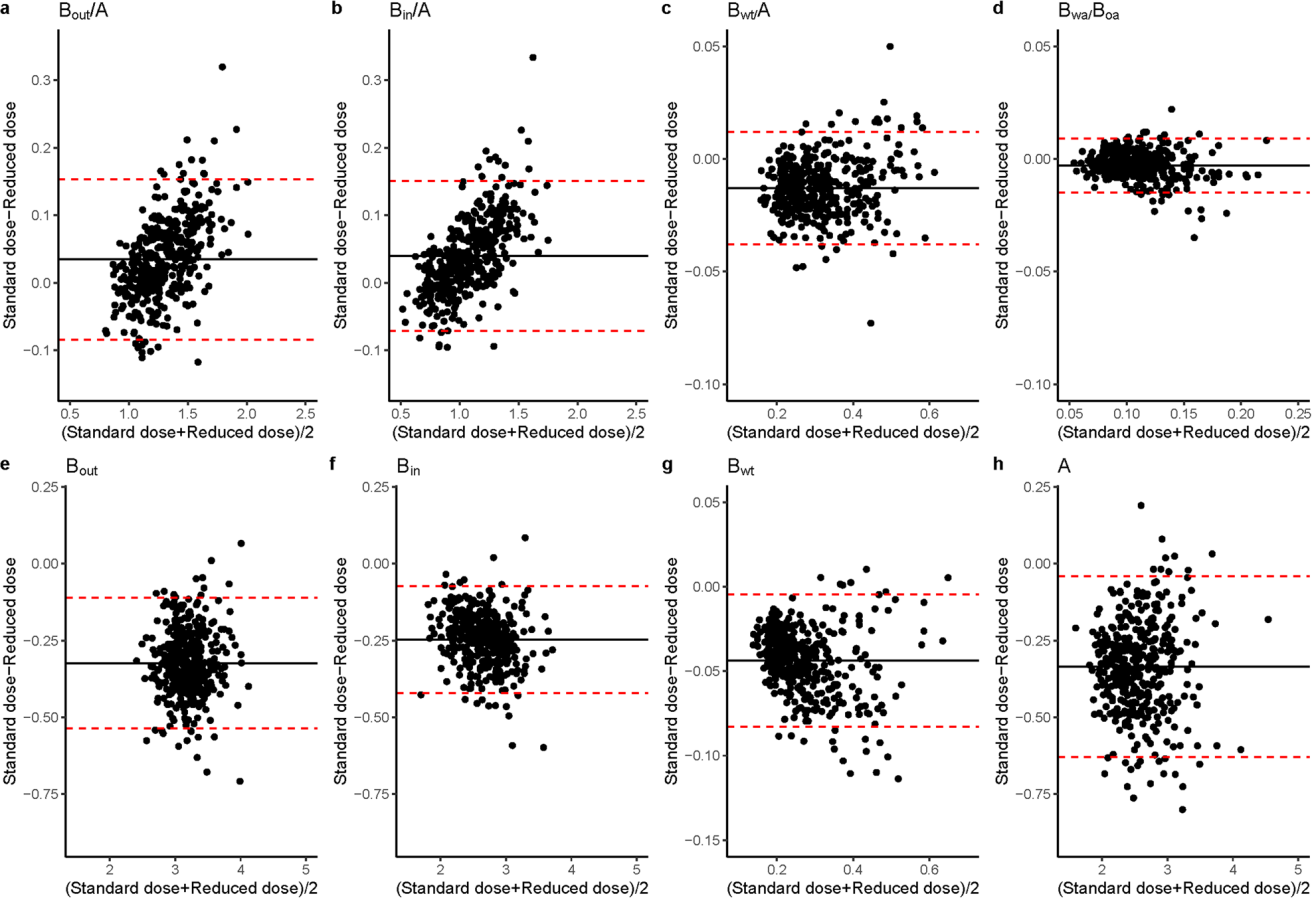
Bland–Altman plot for the difference in BA metrics between FD and RD CT scans with a TLC difference of less than 1%. For this figure, 396 (30%) patients with a TLC difference of less than 1% between FD and RD CTs were selected. Bland–Altman plots were used to illustrate the differences in BA ratios (**a**–**d**) and BA dimensions (**e**–**h**) between FD and RD CT scans. BA, bronchus and artery; *B*_out_, bronchial outer diameter; *B*_in_, bronchial inner diameter; *B*_wt_, bronchial wall thickness; A, artery diameter; *B*_wa_/*B*_oa_, bronchial wall area divided by bronchial outer area

A subgroup of 393 (30%) patients with ΔTLC-CT % < 1% was selected to assess the effect of dose on BA metrics. Differences in BA-metrics between FD and RD scans with ΔTLC-CT% < 1% using Bland–Altman plots are shown in Fig. 4. The biases (limits of agreements) were for *B*_out_/*A* 0.040 (−0.113, 0.193), *B*_in_/*A* 0.043 (−0.111, 0.196), *B*_wt_/*A* −0.012 (−0.073, 0.050), and *B*_wa_/*B*_oa_ −0.002 (−0.024, 0.020), and for *B*_out_ −0.324 (−0.0537, −0.111), *B*_in_ −0.247, (−0.421, −0.072), *B*_wt_ −0.044 (−0.083, −0.0005), and *A* −0.335 (−0.630, −0.040).

## Discussion

In this study, we have demonstrated that inspiratory lung volume of chest CT scans influences BA metrics. Specifically, we observed that small differences in inspiratory lung volume between two CT scans led to minimal variations in BA dimensions and ratios. However, as the difference in inspiratory lung volume between scans increased, we observed corresponding changes in bronchial dimensions, with an increase of *B*_out_/*A* and *B*_in_/*A* and a decrease of *B*_wt_/*A* and *B*_wa_/*B*_oa_.

Our findings indicated that differences in lung volume of less than 10% between two CT scans had limited effect on BA metrics. However, when the difference exceeded 10%, it had a more substantial impact on BA metrics. These findings are in line with earlier publications suggesting that lung volume standardization is important for accurate diagnosis and sensitive monitoring of airway disease [11, 15, 19–21]. In cases where TLC-CT changes by 10% or more between the two CT scans, the observed changes in bronchial dimensions may not accurately reflect changes related to airway disease. For instance, a lower TLC-CT of a follow-up CT scan relative to the baseline CT reduces the number of BA pairs having a *B*_out_/*A* and *B*_in_/*A* above the cut-off value for bronchiectasis. On the other hand, *B*_wt_/*A* and *B*_wa_/*B*_oa_ can be higher suggesting progression in bronchial wall thickening. To better understand the difference in BA-ratio in relation to variations in lung volume, we compared the effect of a 10% variation in lung volume on *B*_out_/*A* of 0.03 in our study to annual changes observed in a longitudinal study of ataluren in 197 cystic fibrosis patients undergoing two chest CT scans over 48 weeks [25]. The observed difference of 0.03 in relation to lung volume in our study was equal to the observed annual progression of 0.03 for *B*_out_/*A* in the ataluren cystic fibrosis population. For the interpretation of the ataluren study, variations in lung volume were taken into account by adjusting TLC-CT in the statistical model. These results reinforce the need to consider lung volume changes when interpreting bronchial measurements, as these variations can impact the diagnosis. Our findings are in line with a previous study [14] in a lung cancer screening population without airflow obstruction where the bronchial lumen diameter and wall thickness were measured in the bronchi of G_5–7_ (which corresponds to G_2–4_ in our study). It was observed that the bronchial lumen diameter was 26% larger and *B*_wt_ was 5% thinner when there was a 100% increase in lung volume [14]. By converting their volume changes from 100% into a 10% increase (i.e., when lung volume changes from 5200 mL into 5720 mL), the lumen diameter would increase by 2.6% (0.026 mm) and wall thickness decrease by 0.5% (0.005 mm), which are comparable to BA-metrics results. Comparable results were also observed in two studies [13, 26] in COPD patients where the bronchial lumen area was measured in selected bronchi between lung volumes at the TLC level and at functional residual capacity. Similar to our findings, it was concluded that the differences in inspiratory lung volume between chest CTs of less than 10% did not substantially affect the subjective assessments of BA dimensions and ratios. Therefore, the diagnostic accuracy and reliability of the BA metrics are not influenced by minor volume changes but can be impacted by significant volume variations, emphasizing the need for volume optimization during routine chest CT acquisition.

For this study, we made use of CTs that were initially collected to study the impact of radiation dose on lung density. In this lung density study, it was shown that adjusting for lung volume significantly improved the reproducibility of lung density measurements for longitudinal analysis [23]. In our study, we investigated the effect of the FD and RD protocol on BA metrics in 30% of patients who had a lung volume difference of less than 1% between the two CT scans. We observed a slightly decreased *B*_out_/*A* and *B*_in_/*A* and increased *B*_wt_/*A* and *B*_wa_/*B*_oa_ for the RD protocol relative to the FD protocol. For example, if a baseline CT is performed using the FD protocol and a follow-up CT employs the RD protocol, the *B*_out_/*A*, and *B*_in_/*A* may be lower at follow-up than expected on the FD CTs, resulting in an underestimation of bronchiectasis. Conversely, this could lead to an overestimation of bronchial wall thickening with higher *B*_wt_/*A* and *B*_wa_/*B*_oa_ on the RD CTs. Therefore, to address the influence of dose when evaluating the impact of lung volume on BA metrics in our study, we included the interaction between dose and lung volume as a covariate in the statistical models. For *B*_wa_/*B*_oa_, the difference between the FD and RD two scans was close to 0 (Fig. 3d). The advantage of *B*_wa_/*B*_oa_ over *B*_wt_/*A* is that this ratio is independent of the bronchial artery diameter. This *B*_wa_/*B*_oa_ measurement bears similarity to %WAT (percentage wall area of the airway area) as reported in several studies [14, 18, 27, 28]. However, the *B*_wa_/*B*_oa_ outcome as assessed by our fully automatic BA method uses the measurements of a large number of BA pairs from the sub-segmental bronchus (G_1_) up to G_6_, in contrast to %WAT which results are in general only obtained from a limited number of measurements of a selected number of bronchi [29]. Therefore, we consider *B*_wa_/*B*_oa_ a robust metric for assessing bronchial wall thickening. For the assessment of bronchial widening, a systematic but comparable difference was observed for both the *B*_out_/*A* and *B*_in_/*A* between FD and RD scans. Our study shows that the dose level only results in small differences in the results of the BA metrics. However, for longitudinal follow-up studies, using a well-standardized scanning protocol throughout the study is advisable to minimize protocol-related variations.

On average, the lung volume levels during CT acquisition were approximately 14% below TLC as calculated from GLI. This discrepancy may be explained by the fact that CTs were taken in the supine position, while the upright position was used in lung function tests for TLC [30]. Furthermore, the lack of breath-hold training before the CT and the variable coaching of breath-hold instructions during the scans, which were collected at 17 different sites, may also have contributed to this discrepancy. Optimizing volume standardization and TLC-CT will likely lead to more consistent and higher-quality results in bronchial measurements and ultimately improve the sensitivity and accuracy of assessing bronchial changes using the BA method for the diagnosis and monitoring of airway disease both for clinical studies and for clinical care.

The limitation of our study lies in its initial design, which focused on assessing the influence of dose on lung density rather than on investigating the influence of lung volume on BA metrics. However, our analysis shows that this design did not significantly affect our findings. Moreover, we studied a selected elderly population with COPD. Further studies are needed to evaluate the influence of lung volume on BA metrics across younger age groups and for healthy subjects or other disease categories. This would also be valuable in validating software-based normalization of bronchial architecture metrics as an alternative method to mitigate the impact of lung volume variations. Furthermore, it is possible that the impact of lung volume on BA metrics in this COPD cohort can be dependent on the severity of emphysema. Further studies are needed to investigate such interaction which can be of importance, especially for longitudinal follow-up studies. However, even in case there is such an interaction between the severity of emphysema, lung volume, and BA metrics, it is unlikely to change our conclusion that lung volume during CT acquisition should be optimized.

In conclusion, our study shows that chest CT BA metrics provide robust quantification for the diagnosis and monitoring of airway disease. However, we observe a difference in TLC larger than 10% between time points would introduce variation in the bronchial dimensions, significantly affecting the BA metrics. To optimize the diagnosis and monitoring of airway disease, it is essential to standardize and optimize inspiratory lung volume during chest CT acquisition. Such standardization will facilitate more reliable comparisons over time, thus improving the accuracy and sensitivity of bronchial change assessments.

## Supplementary information

ELECTRONIC SUPPLEMENTARY MATERIAL

## Abbreviations

A: Artery diameter
BA: Bronchus and artery
*B*_in_: Bronchial inner wall diameter
*B*_out_: Bronchial outer wall diameter
*B*_wa_/*B*_oa_: Bronchial wall area divided by bronchial outer area
*B*_wt_: Bronchial wall thickness
COPD: Chronic obstructive pulmonary disease
FD: Standard full-dose
GLI: Global lung function initiative
RD: Reduced-dose
TLC: Total lung capacity
TLC-CT: Total lung capacity measured on chest CT
ΔTLC-CT: The difference in total lung capacity measured on two chest CTs
ΔTLC-CT%: The difference in total lung capacity as a percentage of the highest lung volume CT
