# Supplementary material for: Effect of inspiratory lung volume on bronchial and arterial dimensions and ratios on chest computed tomography in patients with chronic obstructive pulmonary disease
Source: Eur Radiol. 2024 Nov 29;35(6):2990–8. doi: 10.1007/s00330-024-11126-3 (PMC12081482; doi:10.1007/s00330-024-11126-3)
Supplement: Supplementary file 1 — ELECTRONIC SUPPLEMENTARY MATERIAL [file 330_2024_11126_MOESM1_ESM.docx]

**Appendix**

**Effect of inspiratory lung volume on bronchial and arterial dimensions and ratios on chest computed tomography**

Yuxin Chen, Rudolfs Latisenko, David A Lynch, Pierluigi Ciet, Jean-Paul Charbonnier, Harm A.W.M. Tiddens

**Supplement Methods**

**Protocol breath-hold instructions for COPDGene substudy**

Acquisition parameters have been specified to allow completion of the scan of the whole lung volume in a single breath-hold of less than 10 seconds. Faster scan time can further reduce breath-hold duration and reduce the likelihood of respiratory motion artifacts.

Consistency of lung inflation volume is also critical to lung density measures. The specification is to achieve a difference in lung inflation smaller than 10% of baseline lung inflation volume for longitudinal time points with the goal of achieving greater than 90% of predicted TLC at both time points. To achieve consistency of breath-hold it is essential that the technologist perform consistent coaching of the subject before the CT acquisition (so that the subject is prepared for the voice commands while in the scanner).

Before the scans are acquired, the coordinator (or trained CT technologist) will review the breathing instructions with the participant and emphasize the importance of following them as closely as possible during the actual imaging of the lungs. In summary, the participant will be instructed to inhale deeply and exhale 3 times and then hold their breath two different ways: with the lungs full of air (TLC scan). The technologist or coordinator should visually confirm that the subject is following the breath-hold coaching as intended (see script below). For example, as individual subjects will vary in their respiratory cycle and compliance with commands, it is important for the technologist or coordinator to give sufficient time for the subject to achieve full inspiration with visual confirmation that this is achieved by watching the subject’s chest before CT scanning commences. If additional expiratory scans are performed, please note that the cephalad/caudal coverage of the lungs from apex to base should be adjusted between the TLC and expiratory CT acquisitions to cover the lungs within the limits of the lung apex and base, e.g. no more than 2 cm cephalad to the apical or 5 cm caudal to the basal lung borders.

An additional scout may be acquired between inspiratory and expiratory acquisitions, to prescribe each of the lung volume CT acquisitions separately so as to minimize CT dose to the subject.

To extract the desired information from the CT images, it is very important that the breathing instructions are followed closely. Refer to this publication(1) for further visual and description information on proper breathing instructions.

An example of a breath-hold coaching script is:

● “Take a deep breath in” (watch the chest to ensure deep breathe is achieved)

● “Let it out” (watch chest to insure exhale is achieved)

● “Take a deep breath in” (watch the chest to insure exhale is achieved and timing of breath cycle for the subject)

● “Let it out”

● “Now breathe all the way IN, IN, IN…” (watch to confirm timing and inhalation is fully achieved and chest is still)

● “Keep holding your breath – DO NOT BREATHE”

● Visually confirm inspiratory breath-hold by watching subject’s chest and commence CT scan.

● “Breathe and Relax.”

**Supplement Results**

**Table S1. Mixed-effect model results in segmental generation G_1-6_ to evaluate the influence of TLC-CT on BA-metrics**

| **B_out_/A** | | | |
| --- | --- | --- | --- |
|  | Value | Standard error | P-value |
| (Intercept) | 1.065 | 0.024 | <0.001 |
| **TLC-CT%** | **0.003** | **0.0003** | **<0.001** |
| Dose protocol (reduced dose) | -0.064 | 0.010 | <0.001 |
| TLC-CT% : Dose protocol | 0.0003 | 0.0001 | 0.019 |
| **B_in_/A** | | | |
|  | Value | Standard error | P-value |
| (Intercept) | 0.644 | 0.024 | <0.001 |
| **TLC-CT%** | **0.005** | **0.0003** | **<0.001** |
| Dose protocol (reduced dose) | -0.051 | 0.009 | <0.001 |
| TLC-CT% : Dose protocol | 0.00009 | 0.0001 | 0.403 |
| **Log(B_wt_/A)** | | | |
|  | Value | Standard error | P-value |
| (Intercept) | 0.157 | 0.023 | 0 |
| **TLC-CT%** | **-0.0008** | **0.0003** | **<0.001** |
| Dose protocol (reduced dose) | -0.057 | 0.008 | <0.001 |
| TLC-CT% : Dose protocol | 0.0008 | 0.0001 | <0.001 |
| **Log(B_wa_/B_oa_)** | | | |
|  | Value | Standard error | P-value |
| (Intercept) | -0.221 | 0.023 | 0 |
| **TLC-CT%** | **-0.011** | **0.0003** | **<0.001** |
| Dose protocol (reduced dose) | -0.0005 | 0.007 | 0.46 |
| TLC-CT% : Dose protocol | 0.0005 | 0.00008 | <0.001 |
| **B_out_** |  |  |  |
|  | Value | Standard error | P-value |
| (Intercept) | 3.565 | 0.034 | 0 |
| **TLC-CT%** | **-0.006** | **0.0004** | **<0.001** |
| Dose protocol (reduced dose) | 0.348 | 0.018 | <0.001 |
| TLC-CT% : Dose protocol | -0.0006 | 0.0002 | 0.774 |
| **B_in_** |  |  |  |
|  | Value | Standard error | P-value |
| (Intercept) | 2.285 | 0.035 | 0 |
| **TLC-CT%** | **0.003** | **0.0004** | **<0.001** |
| Dose protocol (reduced dose) | 0.253 | 0.0134 | <0.001 |
| TLC-CT% : Dose protocol | -0.00003 | 0.0002 | 0.861 |
| **Log(B_wt_)** |  |  |  |
|  | Value | Standard error | P-value |
| (Intercept) | -0.315 | 0.031 | <0.001 |
| **TLC-CT%** | **-0.012** | **0.0003** | **<0.001** |
| Dose protocol (reduced dose) | 0.106 | 0.001 | <0.001 |
| TLC-CT% : Dose protocol | 0.0007 | 0.0001 | <0.001 |
| **Log(A)** |  |  |  |
|  | Value | Standard error | P-value |
| (Intercept) | 1.183 | 0.019 | 0 |
| **TLC-CT%** | **-0.004** | **0.0002** | **<0.001** |
| Dose protocol (reduced dose) | 0.141 | 0.009 | <0.001 |
| TLC-CT% : Dose protocol | -0.00001 | 0.0002 | 0.892 |

B_out_ = bronchial outer diameter. B_in_ = bronchial inner diameter. B_wt_ = bronchial wall thickness. A = artery diameter. B_wa_/B_oa_ = bronchial wall area/bronchial outer area. TLC-CT% = total lung capacity measured on inspiratory chest CT scan as a percentage of the predicted value. Note that the coefficient of TLC-CT% represents the mean increase/decrease of BA-metrics for every additional one percentage in TLC-CT% after adjustment of dose protocol. For example, if TLC-CT% increases by 1%, the average B_out_/A would increase by 0.003 after adjustment of dose protocol. By converting the volume increase from 1% into 10%, for BA-ratios, the B_out_/A and B_in_/A would increase by 0.03 and 0.05 and log(B_wt_/A) and log(B_wa_/B_oa_) would decrease by 0.008 and 0.04. For BA-dimensions, the B_in_ would increase by 0.03 and B_out_, log(B_wt_)_,_ and log(A) would decrease by 0.06, 0.12, and 0.04.

**Supplemental figures**

**Figure S1. Flowchart**

**
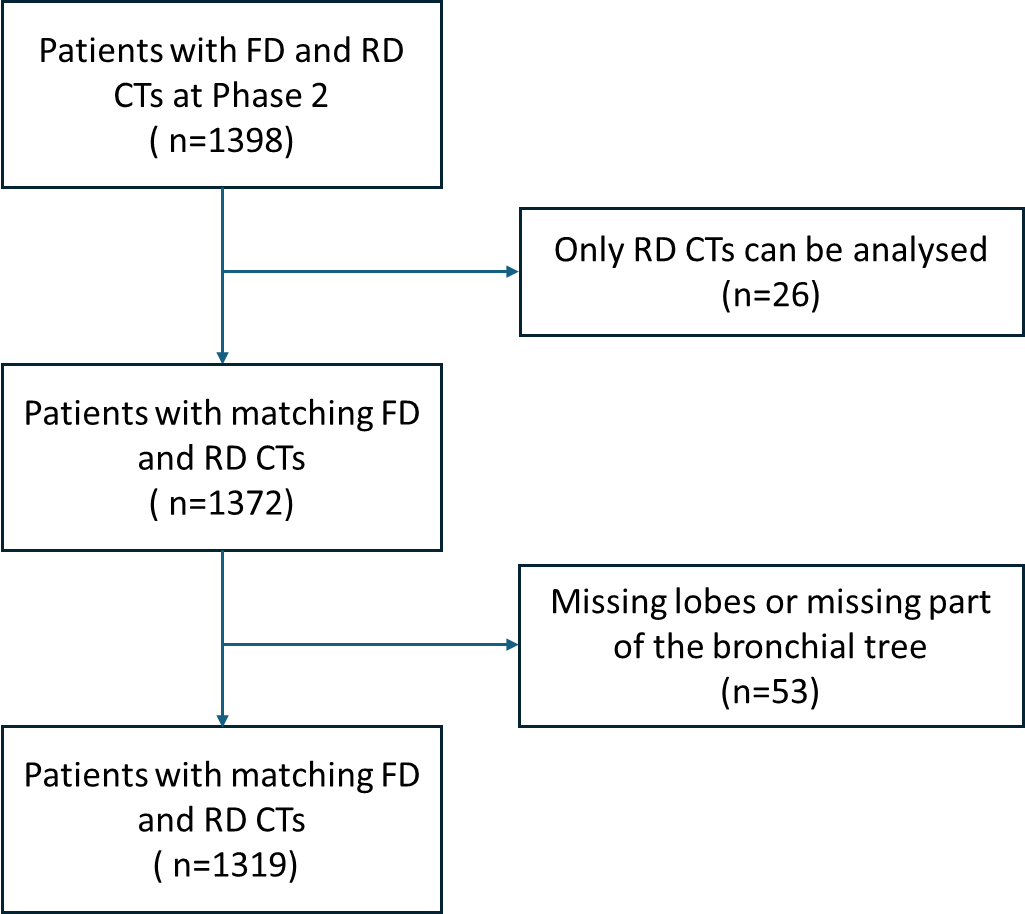
**

FD = fixed dose, RD = reduced dose, CT =computed tomography.

**Figure S2. Difference in total lung capacity between standard full-dose and reduced dose CT scans**

**
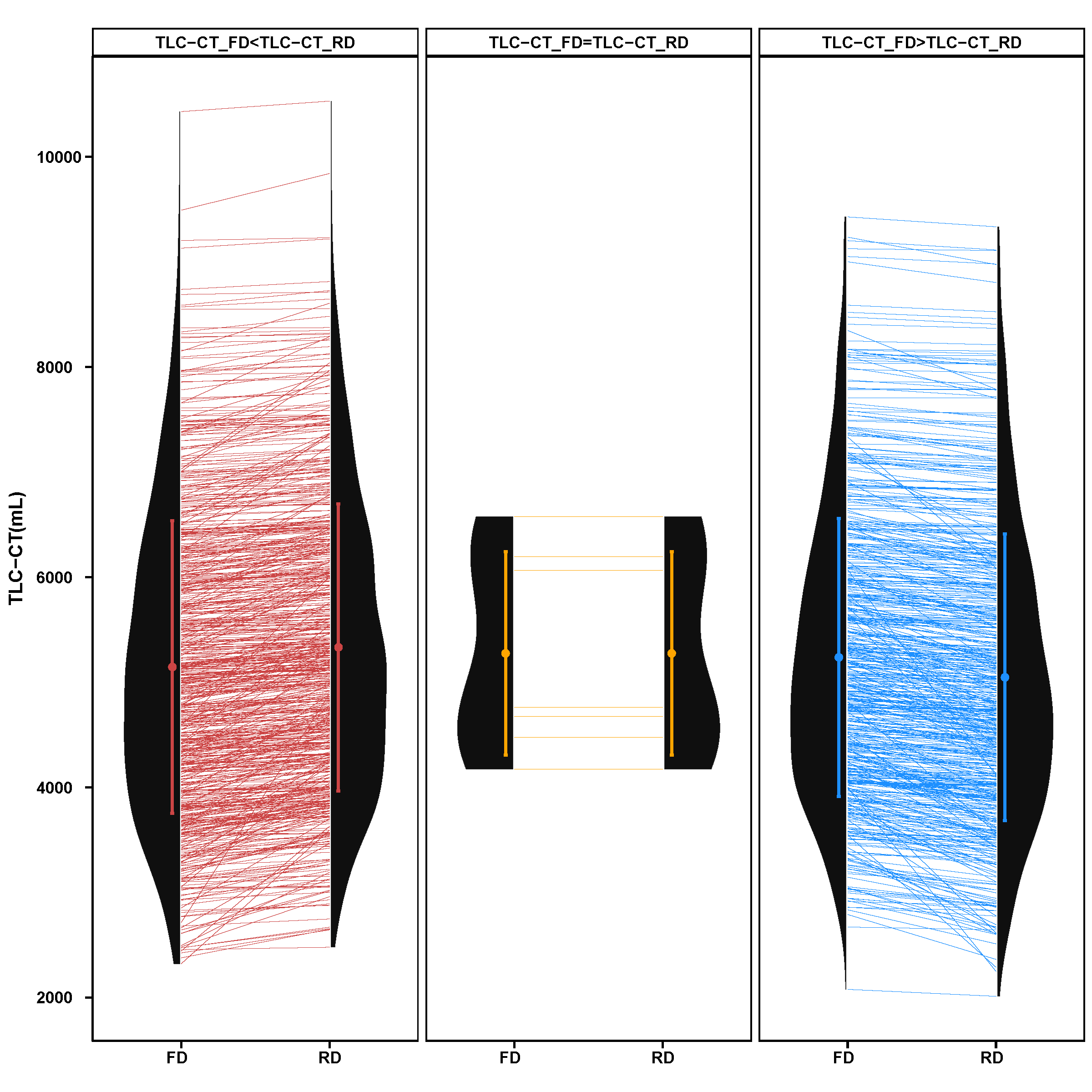
**

The corset plots visualizes the differences in TLC-CT between standard full-dose and reduced-dose CTs. The left panel shows that 701/1319 (53%) patients had lower TLC-CT as measured on standard full-dose CTs than reduced dose CTs; the middle panel showed 7/1319 (<1%) patients had same TLC-CT on both standard full-dose and reduced-dose CTs; the right panel showed 611/1319(46%) patients had higher TLC-CT as measured on reduced-dose CTs than standard full-dose CTs. TLC-CT = total lung capacity measured from chest CT. FD = standard full-dose. RD = reduced-dose.

**Figure S3. Difference in BA-metrics against difference in total lung capacity as determined between standard full-dose and reduced dose CT scans in patietns had lower total lung capacity as measured on standard full-dose CT scans than reduced dose CT scans (n=701)**

**
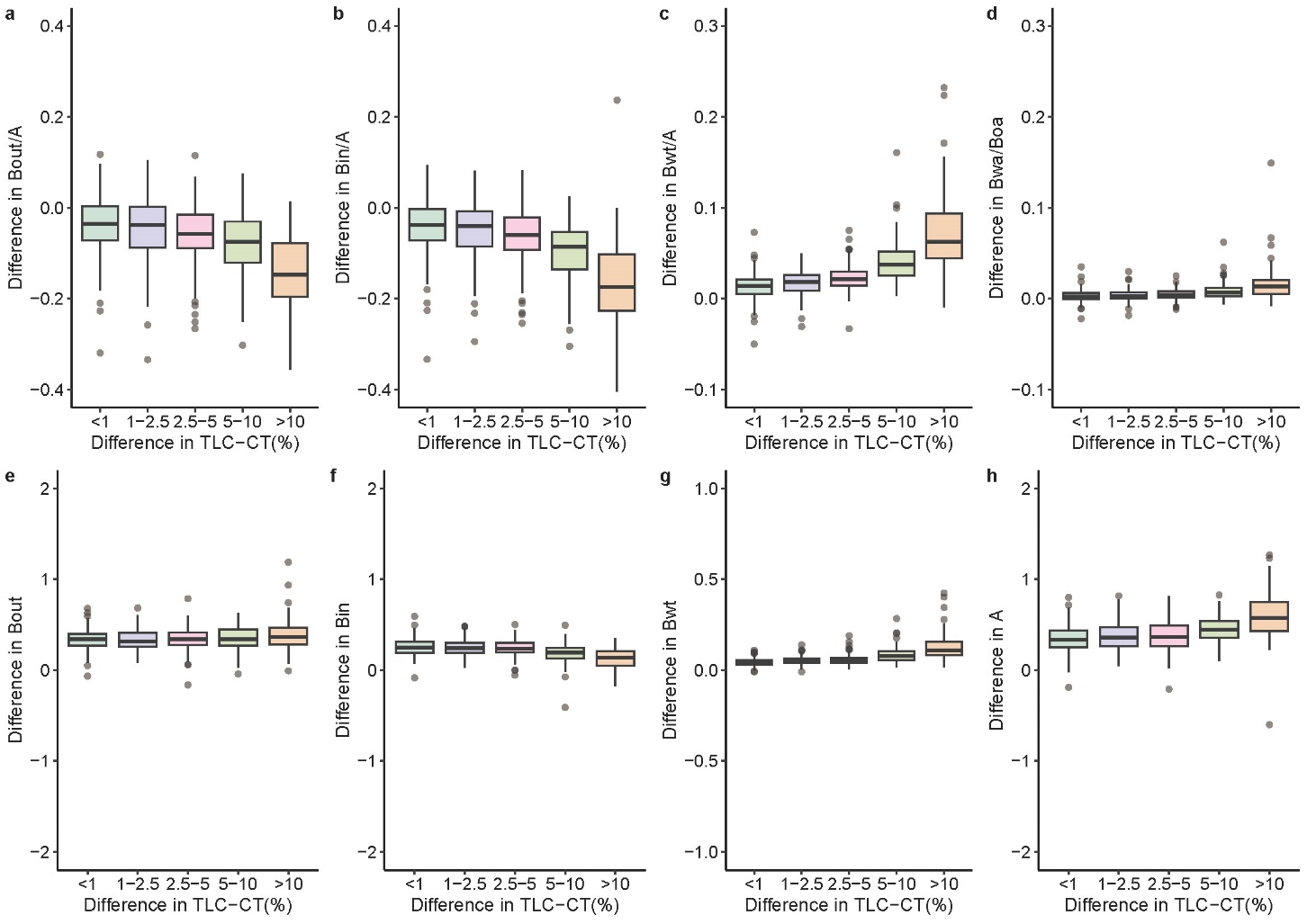
**

Difference in BA-ratios (a-d) and BA-dimensions (e-h) against difference in total lung capacity as determined between standard full-dose and reduced dose CT scans. The difference in TLC-CT was computed between the CT with the highest TLC-CT and lowest TLC-CT (ΔTLC-CT). 701/1319 (53%) patients had lower TLC-CT as measured on standard full-dose CTs than reduced dose CTs. This difference was expressed as a percentage of the highest lung volume CT (ΔTLC-CT %). Differences in BA-metrics was calculated by subtracting the BA-metrics for the CT with the lowest TLC-CT from that of the highest TLC-CT. BA = bronchus and artery. TLC-CT= total lung capacity measured from chest CT. B_out_ = bronchial outer diameter. B_in_ = bronchial inner diameter. B_wt_ = bronchial wall thickness. A = artery diameter. B_wa_/B_oa_ = bronchial wall area divided by bronchial outer area.

**Figure S4. Difference in BA-metrics against difference in total lung capacity as determined between standard full-dose and reduced dose CT scans in patietns had higher total lung capacity as measured on standard full-dose CT scans than reduced dose CT scans (n=611)**

**
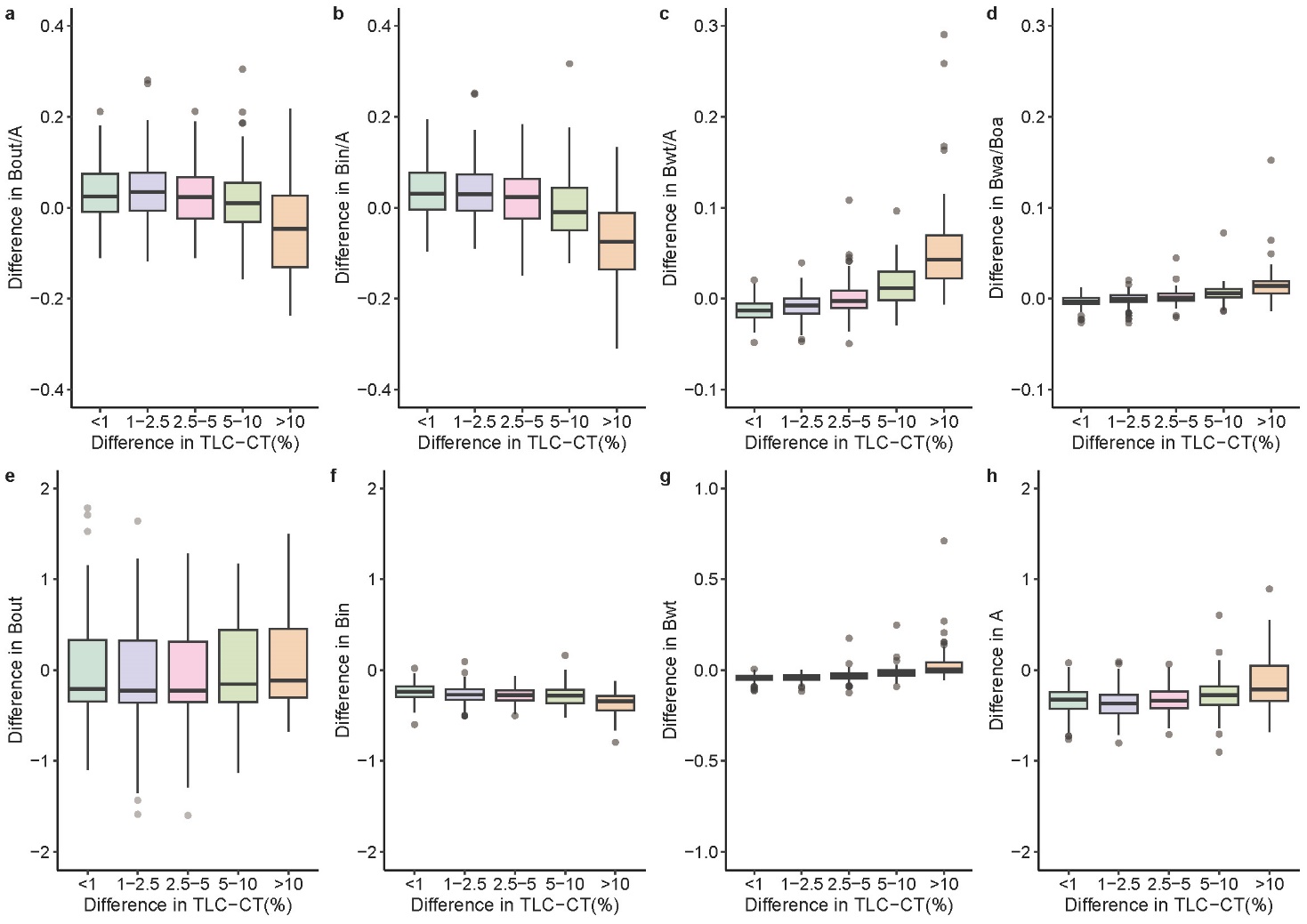
**

Difference in BA-ratios (a-d) and BA-dimensions (e-h) against difference in total lung capacity as determined between standard full-dose and reduced dose CT scans. The difference in TLC-CT was computed between the CT with the highest TLC-CT and lowest TLC-CT (ΔTLC-CT). 611/1319(46%) patients had lower TLC-CT as measured on standard full-dose CTs than reduced dose CTs. This difference was expressed as a percentage of the highest lung volume CT (ΔTLC-CT %). Differences in BA-metrics was calculated by subtracting the BA-metrics for the CT with the lowest TLC-CT from that of the highest TLC-CT. BA = bronchus and artery. TLC-CT= total lung capacity measured from chest CT. B_out_ = bronchial outer diameter. B_in_ = bronchial inner diameter. B_wt_ = bronchial wall thickness. A = artery diameter. B_wa_/B_oa_ = bronchial wall area divided by bronchial outer area.

**Supplemental References**

(1) Regan EA, Hokanson JE, Murphy JR, Make B, Lynch DA, Beaty TH, Curran-Everett D, Silverman EK, Crapo JD. Genetic epidemiology of COPD (COPDGene) study design. Copd 2010;7(1):32-43.
